# Colorectal Cancer: From Pathophysiology to Novel Therapeutic Approaches

**DOI:** 10.3390/biomedicines9121858

**Published:** 2021-12-08

**Authors:** Valeria Barresi

**Affiliations:** Dipartimento di Diagnostica e Sanità Pubblica, Sezione di Anatomia Patologica, Università di Verona, 37134 Verona, Italy; valeria.barresi@univr.it; Tel.: +39-04-5812-4809

According to the Global Cancer Statistics 2020, colorectal cancer (CRC) represents the third most frequent malignancy worldwide, and is the second in terms of mortality [1].

Its higher prevalence in geographic areas with a high human development index is connected with dietary habits such as a high consumption of red meat and alcohol, and with a sedentary lifestyle [1]. Since the early 2000s, screening programs and the consequent early identification and removal of pre-cancerous lesions, together with the shift to a healthier lifestyle, have reduced the frequency of CRC cases in high-incidence areas [2,3]. In addition, the development of targeted therapies has given novel therapeutic opportunities for patients affected by this malignancy [4].

Ongoing scientific research, by increasing the knowledge of the pathogenetic mechanisms and identifying novel prognostic and predictive biomarkers, might lead to a further progressive reduction in the incidence and mortality of CRC.

A relevant concept, which emerged in recent years, is the key role of inflammation in in the tumorigenesis, progression, and metastasization of CRC [5]. The induction of an inflammatory status of the colorectal mucosa is the way in which environmental or dietary habits may trigger colorectal carcinogenesis [5,6]. Indeed, a high consumption of alcohol or red meat may alter the composition of the gut microbiota—so-called dysbiosis—with a decrease in commensal bacterial species (i.e., butyrate-producing bacteria) and the growth of detrimental bacterial strains (i.e., pro-inflammatory opportunistic pathogens) [7]. Aside from a role in the initiation of CRC, dysbiosis seems also to be involved in the resistance to some chemotherapeutic agents, due to its ability to modulate the immune response, and is associated with shorter cancer-specific survival [6,8]. Therefore, probiotics, prebiotics, or antibiotics capable of restoring the normal equilibrium of gut microbiota (eubiosis) could open new scenarios for the prevention or treatment of CRC [6,8].

Notably, assessment of the gut microbiota could represent a non-invasive diagnostic tool for the early identification of CRC [6]. Indeed, some microbial species, including *Escherichia coli*, *Streptococcus gallolyticus*, *Bacteroides fragilis*, *Fusobacterium nucleatum*, and *Enterococcus faecalis*, among others, were found in the stools of patients with colorectal adenoma or carcinoma, but not in heathy subjects [9]. 

The likely role of inflammation in the progression of CRC is also suggested by the prognostic significance of the blood count of neutrophils in patients with this neoplasia [10]. Of note, a high neutrophil-to-lymphocyte ratio (H-NLR) was found to be significantly associated with shorter overall survival in patients with non-metastatic CRC at diagnosis (pathological TNM Stages I and II) [10]. The mechanism by which a high H-NLR could influence disease progression is still to be clarified; however, its association with histopathological features connected with tumor de-differentiation (e.g., poorly differentiated clusters) [10] suggests that an inflammatory status may induce the activation of pathways connected to the epithelial mesenchymal transition in CRC.

The notion that a percentage of CRCs are inflammation-induced has prompted the investigation of the tumorigenic role of some pro-inflammatory proteins, such as the membrane-bound metalloproteinase ADAM17, which induces the release of TNF-α and regulates IL-6 signaling [11].

In spite of the development of novel therapeutic strategies for CRC, there are still several open questions. A dilemma is whether and which patients with non-metastatic CRC could benefit from adjuvant post-surgical therapies. Indeed, the treatment decision regarding patients with CRC is currently based on the pTNM stage, which is regarded as the main prognostic factor. However, a percentage of non-metastatic CRCs unexpectedly progress [12]; therefore, additional prognostic markers are urgently needed to identify high-risk patients who could benefit from adjuvant treatments. In this regard, several histopathological factors, including lympho-vascular invasion, poor tumor differentiation according to the World Health Organization (WHO) grading system, perineural invasion, tumor budding, and poorly differentiated clusters (PDC) are considered high-risk factors for the progression of non-metastatic CRC [13]. A recent consensus on best practice established that pTNM Stage II CRC should be considered at a high risk of progression, even if tumor budding is the only histopathological risk factor present [14].

If confirmed in other studies, H-NLR may also represent a prognostic biomarker of a higher risk of progression in patients with non-metastatic CRC and may therefore be used for the identification of subjects who may benefit from adjuvant treatments [10]. 

Liquid biopsy might also be a promising tool for the identification of CRC patients at a high risk of progression [8,15]. This represents the isolation of cancer-derived components, such as circulating tumor cells (CTC), circulating tumor DNA (ctDNA), microRNAs (miRNAs), long non-coding RNAs (lncRNAs), and proteins, from the peripheral blood or other body fluids [8,15]. Although its use in routine practice is still limited by the lack of validation, the demonstration of cancer-derived components in the blood of patients with non-metastatic CRC may be relevant to identifying patients at a high risk of progression. 

In the last two decades, the discovery of molecular therapeutic targets in CRC allowed the development of several targeted therapies based on the use of monoclonal antibodies [4]. Although these are more effective and display lower toxicity compared with traditional chemotherapy [16], they have not produced a substantial increase in the 5-year survival rate of patients with metastatic (Stage IV) CRC, which is still less than 10% [17]. The failure of targeted therapies may be due to several reasons. First, in most cases, the presence of the target, or of eventual resistance-related mutations, is assessed in the primary tumor and not in the metastases, which actually represent the neoplastic diseases to be treated. Therefore, targeted therapy’s inefficacy may be due to a dissimilarity in the genetic abnormalities between the primary CRC and the matched metastases, as reported in several studies [18]. The discordance between the primary tumor and the metastases may be due to a subclonal evolution during metastasization, or to the genetic heterogeneity of the primary tumor [18]. A study analyzing matched samples showed that the genetic alterations in lymph node metastases reflect those found in the invasive front rather than in the main tumor mass of primary CRC, suggesting that the assessment of molecular targets should be preferentially carried out in this part of the primary tumor [19]. However, discordant alterations may also be present among the different metastases [18].

Another mechanism of drug resistance may also be related to the therapy-induced selection of cancer stem cells, which represent tumor cells that are able to self-renew and to generate tumor cells harboring different genetic alterations [20]. Therefore, understanding their molecular features may be useful for developing therapeutic strategies that are able to target cancer stem cells and to overcome drug resistance.

In conclusion, although the knowledge of the mechanisms underlying the pathogenesis, progression, and metastasization of CRC has greatly expanded in recent decades, many aspects still remain to be clarified. This Special Issue represents a collection of original and review articles focused on recent advances in CRC, providing new insights for future research in this field.
